# Supplementary material for: Herbal Medicine for Postpartum Pain: A Systematic Review of Puerperal Wind Syndrome (Sanhupung)
Source: Healthcare (Basel). 2023 Oct 16;11(20):2743. doi: 10.3390/healthcare11202743 (PMC10606538; doi:10.3390/healthcare11202743)
Supplement: Supplementary file 1 [file healthcare-11-02743-s001.zip › healthcare-2619522-supplementary.pdf]

Supplement Table S1. Search strategies (Search date : 2021.09.03.~2021.09.30.)

Pubmed

	Searches
#1	(Puerperal Wind[Title/Abstract]) OR (Postpartum Wind[Title/Abstract])
#2	((((postpartum period[MeSH Terms]) OR (postpartum[Title/Abstract])) OR (postnatal[Title/Abstract])) OR (puerper*[Title/Abstract])) OR (after childbirth[Title/Abstract])
#3	(((((((((((((((((((((((((pain[MeSH Terms]) OR (myalgia[MeSH Terms])) OR (arthralgia[MeSH Terms])) OR (musculoskeletal pain[MeSH Terms])) OR (Paresthesia[MeSH Terms])) OR (Hypesthesia[MeSH Terms])) OR (pain*[Title/Abstract])) OR (pantalgia[Title/Abstract])) OR (ache*[Title/Abstract])) OR (myalgia[Title/Abstract])) OR (pain*, muscle[Title/Abstract])) OR (arthralgia[Title/Abstract])) OR (pain*, joint[Title/Abstract])) OR (soreness[Title/Abstract])) OR (arthrodynia[Title/Abstract])) OR (bodily pain[Title/Abstract])) OR (Paresthesia*[Title/Abstract])) OR (Dysesthesia*[Title/Abstract])) OR (numbness[Title/Abstract])) OR (tingling[Title/Abstract])) OR (cold hypersensitivity[Title/Abstract])) OR (cold sens*[Title/Abstract])) OR (cold intolerance[Title/Abstract])) OR (hypesthesia*[Title/Abstract])) OR (hypoesthesia*[Title/Abstract])) OR (sensation disorder[Title/Abstract])) OR (sensation dysfunction[Title/Abstract])) OR (sensation loss[Title/Abstract])
#4	#1 OR (#2 AND #3)
#5	((((((((((((((((Medicine, Chinese Traditional[MeSH Terms]) OR (Medicine, Korean Traditional[MeSH Terms])) OR (Medicine, Kampo[MeSH Terms])) OR (Herbal Medicine[MeSH Terms])) OR (Plants, Medicinal[MeSH Terms])) OR (Drugs, Chinese Herbal[MeSH Terms])) OR (herbal medicin*[Title/Abstract])) OR (herbal drug[Title/Abstract])) OR (chinese medicine[Title/Abstract])) OR (korean medicine[Title/Abstract])) OR (japanese medicine[Title/Abstract])) OR (kampo medicine[Title/Abstract])) OR (chinese formula[Title/Abstract])) OR (chinese prescription[Title/Abstract])) OR (chinese decoction[Title/Abstract])) OR (phytomedicine[Title/Abstract])) OR (herb*[Title/Abstract])) OR (botanic*[Title/Abstract])
#6	#4 AND #5

Embase

	Searches
#1	'puerperal wind':ab,ti OR 'postpartum wind':ab,ti
#2	'puerperium'/exp OR postpartum:ab,ti OR postnatal:ab,ti OR puerper*:ab,ti OR 'after

	childbirth':ab,ti
#3	'pain'/exp OR 'myalgia'/exp OR 'arthralgia'/exp OR 'musculoskeletal pain'/exp OR 'paresthesia'/exp OR 'hypesthesia'/exp OR 'dysesthesia'/exp OR 'cold sensitivity'/exp OR pain*:ab,ti OR ache*:ab,ti OR pantalgia:ab,ti OR myalgia:ab,ti OR 'muscle pain*':ab,ti OR arthralgia:ab,ti OR 'joint pain*':ab,ti OR soreness:ab,ti OR arthrodynia:ab,ti OR 'bodily pain':ab,ti OR paresthesia*:ab,ti OR dysesthesia*:ab,ti OR numbness:ab,ti OR tingling:ab,ti OR 'cold hypersensitivity':ab,ti OR 'cold sens*':ab,ti OR 'cold intolerance':ab,ti OR hypesthesia*:ab,ti OR hypoesthesia:ab,ti OR 'sensation disorder':ab,ti OR 'sensation dysfunction':ab,ti OR 'sensation loss':ab,ti
#4	#2 AND #3
#5	#1 OR #4
#6	'chinese medicine'/exp OR 'korean medicine'/exp OR 'kampo medicine'/exp OR 'herbal medicine'/exp OR 'plant medicinal product'/exp OR 'herbaceous agent'/exp OR 'medicinal plant'/exp OR 'chinese herb'/exp OR 'oriental medicine'/exp OR 'herb'/exp OR 'herbal medicine':ab,ti OR 'herbal drug':ab,ti OR 'chinese medicine':ab,ti OR 'korean medicine':ab,ti OR 'japanese medicine':ab,ti OR 'kampo medicine':ab,ti OR 'chinese formula':ab,ti OR 'chinese prescription':ab,ti OR 'chinese decoction':ab,ti OR phytomedicine:ab,ti OR 'herbal preparation':ab,ti OR botanic:ab,ti
#7	#5 AND #6

Cochrane library

	Searches
#1	puerperal wind:ti,ab OR postpartum wind:ti,ab
#2	[mh "postpartum period"] OR postpartum:ti,ab OR postnatal:ti,ab OR puerper*:ti,ab OR "after childbirth":ti,ab
#3	[mh pain] OR [mh myalgia] OR [mh arthralgia] OR [mh "musculoskeletal pain"] OR [mh Paresthesia] OR [mh Hypesthesia] OR pain*:ti,ab OR ache*:ti,ab OR pantalgia:ti,ab OR myalgia:ti,ab OR "muscle pain*":ti,ab OR arthralgia:ti,ab OR "joint pain*":ti,ab OR soreness:ti,ab OR arthrodynia:ti,ab OR "bodily pain":ti,ab OR Paresthesia*:ti,ab OR Dysesthesia*:ti,ab OR numbness:ti,ab OR tingling:ti,ab OR "cold hypersensitivity":ti,ab OR "cold sens*":ti,ab OR "cold intolerance":ti,ab OR Hypesthesia*:ti,ab OR hypoesthesia*:ti,ab OR "sensation disorder":ti,ab OR "sensation dysfunction":ti,ab OR "sensation loss":ti,ab
#4	#1 OR (#2 AND #3)

#5	[mh "Medicine, Chinese Traditional"] OR [mh "Medicine, Korean Traditional"] OR [mh "Medicine, Kampo"] OR [mh "Herbal Medicine"] OR [mh "Plants, Meidicinal"] OR [mh "Drugs, Chinese Herbal"] OR "Herbal Medicine":ti,ab OR "herbal drug":ti,ab OR "chinese medicine":ti,ab OR "korean medicine":ti,ab OR "japanese medicine":ti,ab OR "kampo medicine":ti,ab OR "chinese formula":ti,ab OR "chinese prescription":ti,ab OR "chinese decoction":ti,ab OR phytomedicine:ti,ab OR herb*:ti,ab OR botanic*:ti,ab
#6	#4 AND #5

CNKI

	Searches
#1	((TI = '产后风' OR TI = '产后身痛' OR TI = '产后疼痛' OR TI = ‘产后遍身疼痛’ OR TI = '产后身体疼痛' OR TI = '产后全身痛' OR TI = '产后全身疼痛' OR TI = '产后肢体疼痛' OR TI = '产后全身酸痛' OR TI = '产后关节痛' OR TI = '产后关节疼痛' OR TI = '产后痹' OR (TI = '产后' AND TI = '痹证') OR (TI = '产后' AND TI = '痹症') OR (TI = '产后' AND TI = '麻木') OR (TI = '产后' AND TI = '肢麻') OR (TI = '产后' AND TI = '感觉异常') OR (TI = '产后' AND TI = '感觉减退') OR (TI = '产后' AND TI = '无感觉') OR (TI = '产后' AND TI = '感觉障碍') OR (TI = '产后' AND TI = '重着') OR (TI = '产后' AND TI = '酸重') OR (TI = '产后' AND TI = '酸困') OR (TI = '产后' AND TI = '酸懒') OR (TI = '产后' AND TI = '恶風') OR (TI = '产后' AND TI = '冷过敏') OR (TI = '产后' AND TI = '冷超敏性') OR (TI = '产后' AND TI = '寒冷超敏反应') OR (TI = '产后' AND TI = '冷感') OR (TI = '产后' AND TI = '不耐寒') OR TI = 'puerperal wind' OR TI = 'postpartum wind' OR TI = 'postpartum pain' OR TI = 'postpartum pantalgia' OR TI = 'pantalgia after childbirth' OR TI = 'postpartum arthralgia' OR TI = 'postpartum joint pain' OR TI = 'postpartum myalgia' OR TI = 'postpartum muscle pain' OR TI = 'postpartum ache' OR TI = 'postpartum dysesthesia' OR TI = 'postpartum hypesthesia' OR TI = 'postpartum cold intolerance') AND (TI = '中医' OR TI = '中药' OR TI = '汤' OR TI = '丸' OR TI = '散' OR TI = '方' OR TI = '颗粒' OR TI = '胶囊' OR TI = '自拟' OR TI = '中西医结合' OR TI = 'Herbal medicine' OR TI = 'Traditional chinese medicine' OR TI = 'Pill' OR TI = 'Powder' OR TI = 'Prescription' OR TI = 'Capsule' OR TI = 'Decoction' OR TI = 'Formula')) OR ((AB = '产后风' OR AB = '产后身痛' OR AB = '产后疼痛' OR AB = ‘产后遍身疼痛 OR AB = '产后身体疼痛' OR AB = '产后全身痛' OR AB = '产后全身疼痛' OR AB = '产后肢体疼痛' OR AB = '产后全身酸痛' OR AB = '产后关节痛' OR AB = '产后关节疼痛' OR (AB = '产后' AND AB = '痹证') OR (AB = '产后' AND AB = '痹症') OR (AB = '产后' AND AB = '麻木') OR (AB = '产后' AND AB = '肢麻') OR (AB = '产后' AND AB = '感觉异常') OR

	(AB = '产后' AND AB = '感觉减退') OR (AB = '产后' AND AB = '无感觉') OR (AB = '产后' AND AB = '感觉障碍') OR (AB = '产后' AND AB = '重着') OR (AB = '产后' AND AB = '酸重') OR (AB = '产后 ' AND AB = '酸困') OR (AB = '产后' AND AB = '酸懒') OR (AB = '产后' AND AB = '恶風') OR (AB = '产后' AND AB = '冷过敏') OR (AB = '产后' AND AB = '冷超敏性') OR (AB = '产后' AND AB = ' 寒冷超敏反应') OR (AB = '产后' AND AB = '冷感') OR (AB = '产后' AND AB = '不耐寒') OR AB = 'puerperal wind' OR AB = 'postpartum wind' OR AB = 'postpartum pain' OR AB = 'postpartum pantalgia' OR AB = 'pantalgia after childbirth' OR AB = 'postpartum arthralgia' OR AB = 'postpartum joint pain' OR AB = 'postpartum myalgia' OR AB = 'postpartum muscle pain' OR AB = 'postpartum ache' OR AB = 'postpartum dysesthesia' OR AB = 'postpartum hypesthesia' OR AB = 'postpartum cold intolerance') AND (AB = '中医' OR AB = '中药' OR AB = '汤' OR AB = '丸' OR AB = '散' OR AB = '方' OR AB = '颗粒' OR AB = '胶囊' OR AB = '自拟' OR AB = '中西医结合' OR AB = 'Herbal medicine' OR AB = 'Traditional chinese medicine' OR AB = 'Pill' OR AB = 'Powder' OR AB = 'Prescription' OR AB = 'Capsule' OR AB = 'Decoction' OR AB = 'Formula'))

CiNii

	Searches
#1	(産後風 OR 産後の痛 OR 産後の疼痛 OR 産後の関節痛 OR 産後の激痛 OR 産後の苦痛 OR 産後の筋痛症 OR 産後 痺 OR 産後 麻木 OR 産後 肢麻 OR 産後 異常感覚 OR 産後 知覚異常 OR 産後 感覚鈍麻 OR 産後 しびれ OR puerperal wind OR postpartum wind OR postpartum pantalgia OR postpartum arthralgia OR postpartum joint pain OR postpartum myalgia OR postpartum muscle pain OR postpartum ache OR Postpartum Paresthesia OR Postpartum Dysesthesia OR Postpartum Hypesthesia OR Postpartum numbness OR Postpartum cold intolerance) AND (漢方薬 OR 伝統医学 OR 漢方医学 OR 韓国医学 OR 東洋医学 OR 代替医療 OR 補完医学 OR 製剤 OR 剤 OR 中药 OR 湯 OR 丸 OR 散 OR 方 OR 颗粒 OR Herbal medicine OR Chinese medicine OR Korean medicine OR Japanese medicine OR kampo OR pill OR powder OR prescription OR capsule OR decoction OR formula)

OASIS

	Searches
#1	(산후풍 ǀ 산후신통 ǀ 산후통) (한약 ǀ 한의학 ǀ 한의약 ǀ 중약 ǀ 중의학 ǀ 중의약 ǀ 처방 ǀ 湯 ǀ 丸 ǀ 散 ǀ 劑 ǀ 方 ǀ 과립)

ScienceOn

	Searches
#1	산후풍 I 산후신통 I 산후통 I "puerperal wind" I "postpartum wind" I "postpartum pain"<br>한약 I 한의학 I 한의약 I 중약 I 중의학 I 중의약 I 처방 I 湯 I 丸 I 散 I 劑 I 方 I 과립

Kiss

	Searches
#1	산후풍 I 산후신통 I 산후통 I "puerperal wind" I "postpartum wind" I "postpartum pain"<br>한약 I 한의학 I 한의약 I 중약 I 중의학 I 중의약 I 처방 I 湯 I 丸 I 散 I 劑 I 方 I 과립

KMBASE

	Searches
#1	(((((([ALL=산후풍] OR [ALL=산후신통]) OR [ALL=산후통]) OR [ALL=puerperal wind]) OR [ALL=postpartum wind]) OR [ALL=postpartum pain]) AND ((((((((((([ALL=한약] OR [ALL=한의학]) OR [ALL=한의약]) OR [ALL=중약]) OR [ALL=중의학]) OR [ALL=중의약]) OR [ALL=처방]) OR [ALL=湯]) OR [ALL=丸]) OR [ALL=散]) OR [ALL=劑]) OR [ALL=方]) OR [ALL=과립]))

한약, 한의학, 한의약, Korean medicine; 중약, 중의학, 중의약, Chinese medicine; 처방, Prescription; 湯, Decoction; 丸, pill, 散, powder; 劑, Prescription; 方, formula; 과립, Capsule.
